# Bridging the Gap: A Pilot Study of a Health Care Transition Podcast Curriculum and Standardized Patient Case for Graduate Medical Education Trainees

**DOI:** 10.7759/cureus.72018

**Published:** 2024-10-21

**Authors:** Colby D Feeney, Rebecca E Sadun, Alison Manning, Jane V Trinh

**Affiliations:** 1 Department of Medicine, Duke University School of Medicine, Durham, USA; 2 Department of Pediatrics, Duke University School of Medicine, Durham, USA; 3 Department of Psychiatry and Human Behavior, The Warren Alpert Medical School of Brown University, Providence, USA

**Keywords:** adolescent and young adults, curriculum development and evaluation, health care transitions, med-peds, objective structured clinical examination (osce), residency curriculum

## Abstract

Introduction

Pediatric to adult health care transition (HCT) is critical to maintaining the health and wellness of patients, and pediatric and adult providers often do not feel prepared to shepherd patients through this process.

Methods

We designed an HCT curriculum consisting of nine podcasts paired with existing ambulatory experiential learning opportunities for internal medicine-pediatric residents (n=6). Before and after the curriculum we evaluated resident HCT self-assessment and resident performance working with a standardized patient (SP) and standardized parent in a novel objective structured clinical examination (OSCE) station designed to assess HCT skills.

Results

Residents improved in all HCT self-assessment goals (Likert 1-5; average score increasing from 2.4 to 3.93; p= 0.0002) and in their overall OSCE performance (Likert 1-5; average score increasing from 2.6 to 4.1; p=0.002).

Conclusion

By combining portable didactic educational materials with intentional experiential learning opportunities, the HCT curriculum herein described improved residents’ knowledge and skills related to helping adolescent and young adult patients through HCT. This curriculum could be easily adapted for implementation at other institutions and across disciplines. In addition, we offer a standardized patient case for use in assessing HCT skills.

## Introduction

Health care transition (HCT) is the process of gaining independence in managing one’s own medical care [1,2]. Ideally, this process begins in early adolescence and continues after transfer, often continuing throughout early adulthood (18-25 years of age). During this time period, patients experience not only a change in medical care, but often simultaneous social changes to their family support and structure, educational and vocational standing, living situation, and insurance status [3]. The timing of these changes is coupled with worsening disease and comorbidities for many childhood-onset chronic conditions and creates a very high-risk period for the patient’s health. Studies reveal that a lack of structured HCT interventions can lead to medical complications, limitations in health and well-being, problems with treatment and medication adherence, discontinuity of care, patient dissatisfaction, higher emergency department and hospital use, and higher costs of care [4-6].

Only 17% of patients with special health care needs report receiving HCT preparation [7,8]. Providers caring for patients during the transitional years report little training in transition skills, low confidence in their abilities to help patients with transition, and the desire to learn more about specific disease processes as well as the behavioral stages of young adult development [9,10]. Studies show that both pediatric and adult providers feel inadequately prepared for this process. It is imperative that pediatric, internal medicine, and family medicine trainees receive adequate education and guidance on how to ensure a safe and timely transition for their patients. It is similarly essential that trainees understand health care transition in order to successfully integrate patients into adult care, especially for the large number of patients who have not received HCT counseling [11].

In 2018, our combined internal medicine-pediatrics (med-peds) residency program participated in a national quality improvement collaborative with 20 other med-peds programs to increase the number of health care providers who are able to provide comprehensive care in healthcare transition [12]. We completed a program inventory of current transition training opportunities in our curriculum, as well as objective mapping to evaluate where specific transition goals are taught during training. We found that many objectives were not directly addressed during training and were largely based on unpredictable clinical experiences. Following this needs assessment, we reviewed existing studies of HCT curricula and aimed to create a curriculum that incorporated a multimodal approach to content delivery, built on existing experiential learning opportunities, and included the use of podcasts to deliver educational content [13,14]. We aimed to have the podcasts be brief and focused to enhance knowledge retention [15]. As previous curricula have proven to improve learner attitudes and knowledge of HCT, we aimed to assess improvement in HCT skills through the use of a novel standardized patient (SP) case [16-19]. Skills assessment was undertaken with the goal of assessing the curriculum and providing low-stakes, formative feedback to the trainee.

## Materials and methods

Study objectives

We aimed to assess the impact of a podcast-based health care transition curriculum on the knowledge, skills, and attitudes of residents. Specifically, we gathered resident feedback and measured podcast completion rates to understand resident satisfaction with the podcast curriculum. To assess the effectiveness of the curriculum, we measured residents' self-assessed health care transition skills before and after the curriculum in addition to assessing their performance on an objective structured clinical examination (OSCE) before and after the curriculum.

Curriculum design

Core content areas in the curriculum (supplementary material) were selected by a group of transition experts who also created the podcasts (supplementary material) with the residency leadership team. Content experts included physicians in primary care, hospital medicine, adolescent medicine, and combined sub-specialties, as well as a clinical social worker and a family partner. Topics included the impact of transition on the patient, cultural differences between pediatric and adult care, transition readiness assessment, adolescent and young adult development, mental health concerns, health insurance, educational and vocational needs, and guardianship and other legal issues. Podcast speakers used a template to maintain structure among various speakers. A total of nine podcasts were recorded with an average length of 17 minutes per podcast. The topics discussed in the podcasts were mapped and paired with the various existing clinical experiences. We also assigned supplemental experiential learning tasks, for example, performing a transition readiness assessment of a patient, viewing a transitional school website, talking with the clinical social worker about community resources, and reviewing relevant articles. The supplemental learning tasks were completed during the clinic session or asynchronously on their own, depending on the activity (Table 1). 

**Table 1:** Podcasts Table of Contents SBP = systems-based practice; MK = medical knowledge; PC = patient care; PROF = professionalism; ICS = interpersonal communication skills; SDOH = social determinants of health; PHQ2 = patient health questionnaire-2 ^a^ ACGME. Milestones Supplemental Guide: Internal Medicine. (2020). ^b^ ACGME. Milestones Supplemental Guide: Pediatrics. (2021). Curriculum Goals & Objectives available in the Appendices. Podcasts are on Warpwire and can be accessed at the following link: https://warpwire.duke.edu/w/9d8DAA/

Podcast Topic	Application-(ex: Quiz, Teacher Prompts, discussion, patient application)	Learning Objective(s) (as listed in curriculum)	Competencies (M=Medicine and P=Pediatric)			Additional Resources
			Milestones 1.0	Milestones 2.0 ^a,b^		
Curriculum introduction	Access curriculum materials on Box	N/A	N/A		N/A	Curriculum materials and outline of objectives and content
Transition: define and describe impact/outcomes	Identify patients of transitional age in practice	A1, A5, A6	M-SBP4, P-PC3		M-SBP2, P-SBP4	Gottransition.org, summary paper
Cultural differences between pediatric and adult care	Talk with one patient and family about transition	A4, A5, A6, A7, A8	M-SBP4, P-SBP1,		M-SBP2, P-SBP3	Read Hart. Pediatrics, 2019. PMID: 31676681
Transition readiness/TRAQ	Administer the TRAQ to one patient and discuss findings with preceptor	A2, A3, A7, A8, B1	M-SBP2, P-SBP2		M-SBP2, P-SBP2	Transition Readiness Assessment Questionnaire (TRAQ)
Adolescent and young adult development	Perform mental health screening on patient and discuss with preceptor	B1, B2, B5	M-MK1, P-ICS2,		M-MK1, P-ICS1	Suggested reading
Mental health disorders	Administer a PHQ2 and analyze the results	B3, B6	M-MK1		M-MK1	Websites included in transcript; Safety plan
Young adult insurance	Discussion with preceptor or completion of SDOH tab in electronic health record	C1, C2	M-PC2, M-SBP3, M-SBP4, P-PC3, P-PC5,		M-PC5, M-SBP2, M-SBP3, P-SBP4, P-PC5	List of community resources
Young adult educational and vocational needs and community resources	Discussion with clinic social work	B6	M-SBP1, M-ICS2		M-SBP2, M-ICS2	Durham Public Schools Transition Schools
Financial and legal issues/guardianship and supported decision making	Discussion with preceptor	B4	M-SBP1, M-ICS2, P-SBP3		M-SBP2, M-ICS2, P-SBP3, P-ICS2	Discussion with preceptor

Curriculum implementation

The overarching transition curriculum was designed for the second and third years of our four-year med-peds training program through implementation during a four-week ambulatory rotation block which our residents complete twice: once during the second half of their second post-graduate year (PGY-2), and once during the first half of their PGY-3 year. The didactic podcast curriculum was provided to all of our med-peds residents during their PGY-3 ambulatory rotation.

The clinical experiences in the ambulatory rotation focused on specialties that treat patients over the lifespan; we capitalized on our network of med-peds subspecialists to increase exposure in caring for patients transitioning from pediatric medicine to adult medicine. At the time of our transition curriculum pilot, residents attended a transitional sickle cell clinic, an adult congenital heart disease clinic, adult and pediatric rheumatology clinics, and a transitions primary care clinic. All residents attended all clinics and followed the same schedule in sequential months (one resident on the rotation per month during the six-month pilot). Each resident’s exposure to the clinical experience was identical. We asked residents to listen to the podcasts prior to specific clinical experiences and assigned a clinical application task directly related to what they had learned that week in the podcast.

Curriculum evaluation and learner assessment

We evaluated both process measures (percentage of podcasts completed by each learner and qualitative transition curriculum feedback) and outcome measures (self-assessment scores and SP skills assessment scores). We collected pre- and post-curriculum self-assessments on resident HCT skills using a tool from the HCT Collaborative. Residents reported their self-rated confidence level in transition skills, including helping youth with special health care needs, identifying development and psychosocial challenges during transition, recognizing the impact of insurance policies and social services as patients age into adulthood, addressing the educational and vocational needs of youth with special health care needs, and applying transition health care systems knowledge to improve transition policies. Responses were measured on a five-point Likert scale with 1= having no knowledge or skills and 5 being expert level.

**Figure 1:**
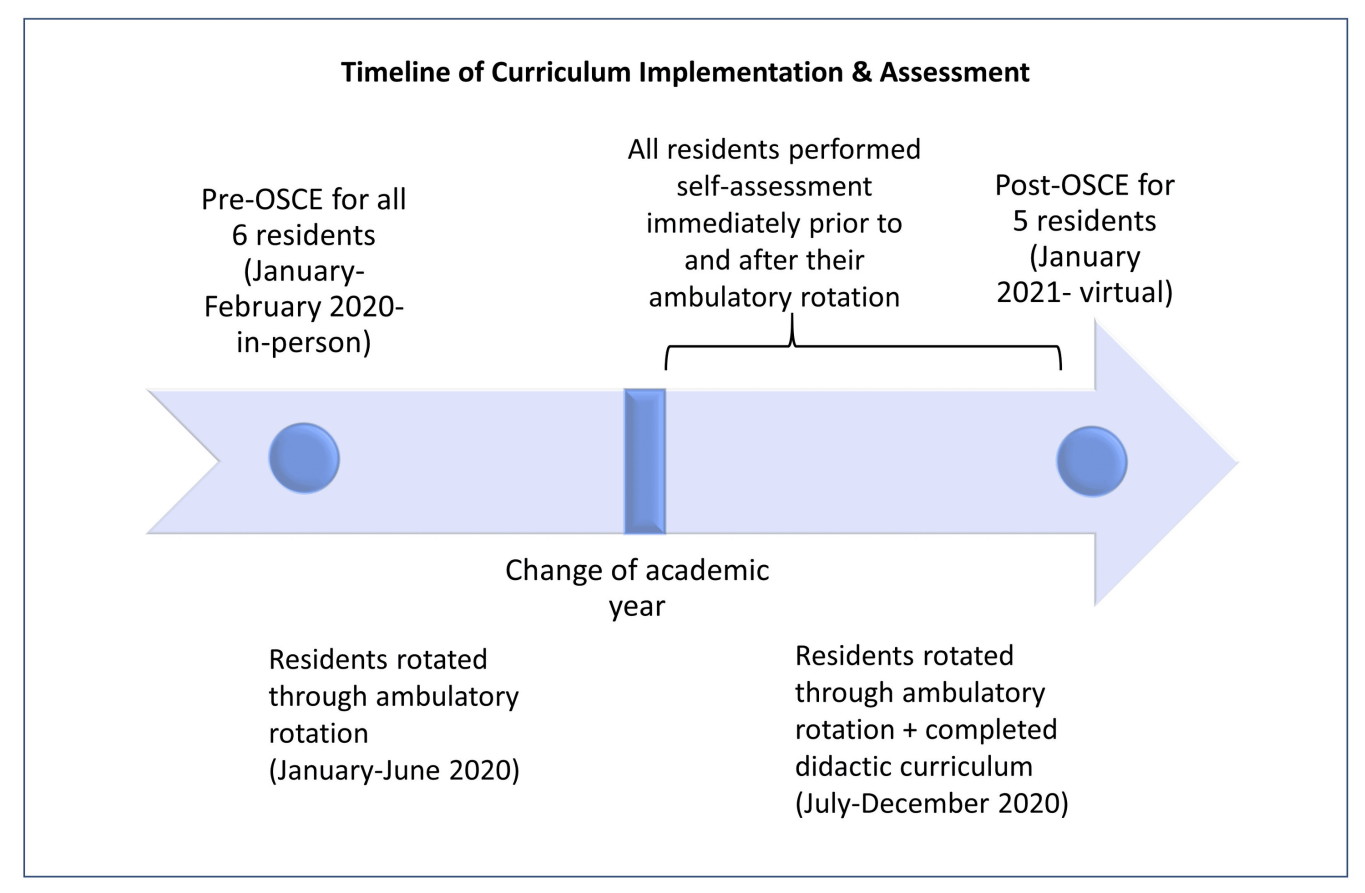
Curriculum Timeline OSCE = objective structured clinical examination

We also performed pre-curriculum and post-curriculum objective structured clinical examinations (OSCE). Of note, the onset of the COVID-19 pandemic occurred after pre-curriculum assessments but before residents’ completion of the curriculum; therefore, the pre-curriculum OSCE was performed in person in early 2020, whereas the post-curriculum OSCE was performed through a virtual platform, mimicking a telehealth visit, approximately one year after the pre-curriculum OSCE (Figure 1). The goals, SP scenario, and basic structure of the OSCE were the same across the two formats, with a few logistic adjustments made to accommodate a virtual platform: for example, whereas the OSCE instructions were posted on the clinic room door for the in-person OSCE, and the standardized patient’s (SP’s) completed Transition Readiness Assessment Questionnaire (TRAQ) was on a clipboard for the in-person visit, the instructions and TRAQ were provided to residents via screen sharing for the virtual, telehealth OSCE. In both scenarios, residents were provided a brief medical history explaining that the patient has a history of asthma and severe peanut allergy; residents were told that the physical examination had already been performed and was within normal limits. 

Each resident was given 15 minutes with the patient, and a three-minute warning was provided prior to the end of the session. Residents were given four tasks for the patient encounter: (1) take an appropriate history, including the interval history and social history; (2) assess the patient’s transition readiness and help the patient set self-management goals; (3) Identify obstacles to transition, including potential social and financial challenges, and offer resources where appropriate; and (4) discuss transition and transfer, and help the young adult and her mother understand what those words mean, why they are important/relevant, what a timeline might look like, and what to expect from adult care.

One faculty facilitator observed each OSCE and provided immediate formative feedback on the different components assessed. The facilitator timed and audio-recorded the session for later evaluation. OSCE performance was assessed by two independent, blinded assessors using a pre-specified tool that included a global assessment of HCT transition skills during the visit, as well as five specific transition skills, each of which was rated on a 1-5 Likert scale using behavioral anchors to assess each domain (Table 2).

**Table 2:** Direct Observation Rating Scale for Transition Skills TRAQ: Transition Readiness Assessment Questionnaire

Direct Observation Rating Scale for Transition Skills				
Resident Name:		Evaluator Name:		Date:
ITEM 1: PLACES PATIENT IN PRIMARY ROLE & USES PARENT FOR CORROBORATION				
1	2	3	4	5
Rarely addresses questions to the patient.		Addresses at least half of the questions to the patient.		Makes it clear the patient should answer first.
Rarely makes eye contact with the patient when providing explanations of instructions.		Makes eye contact with patient much of the time when providing explanations or instructions.		Tactfully reminds parent as needed.
Allows parent to dominate the conversation and run the visit.		Does not explicitly encourage the patient to answer first.		Addresses most of the questions to the patient and makes eye contact with patient the majority of the time.
		Does not effectively look to patient for corroboration.		Effectively elicits corroboration from & involves parent.
Comments:				
ITEM 2: ASSESSES TRANSITION SKILLS				
1	2	3	4	5
Assesses no TRAQ skills	Assesses 1 TRAQ skill	Assess 2+ TRAQ skills	Assesses 2+, deep in 1+, setting at least 1 goal	Assesses 2+ deeply, with 1+ goals or assess 1+ deeply with 2+ goals
Uses TRAQ: Yes/No				
Skills Checklist (circle all that apply): Knows meds/doses, can refill meds, knows what to do for side effects, makes appointments, keeps track of appointments, arranges transportation, calls for problems, manages health insurance, manages money/budget, fills out medical forms, answers doctors' questions, asks questions at appointments				
Comments:				
ITEM 3: ASKS PARENT TO LEAVE ROOM FOR SOCIAL HISTORY & ASSURES PATIENT OF CONFIDENTIALITY				
1	2	3	4	5
Does not ask parent to leave the room.		Asks parent to leave the room, but does not obtain a sexual and drug history under confidentiality.		Obtains sexual, contraceptive, and drug history with parent out of the room.
		Does not explain why the parent should leave the room or explains only partially/poorly.		Skillfully explains why the parent should leave the room.
				Effectively assures patient of confidentiality.
Comments:				
ITEM 4: ASSESSES BARIERS TO TRANSITION & ADHERENCE				
1	2	3	4	5
Does not ask about insurance, finances or transportation.		Assesses 2 potential barriers.	Assesses 3+ barriers.	Skillfully assesses insurance status and 2+ additional potential barriers, offering resources.
Does not assess whether religious beliefs or cultural traditions impact health beliefs.				
Does not determine the patient's level of education or social circumstances.				
Comments:				
ITEM 5: DISCUSSES TRANSITION AND TRANSFER				
1	2	3	4	5
Does not correctly define either transition or transfer.	Defines transition or transfer, correctly, but not both.	Defines both correctly.	Explains both well.	Skillfully explains transition & transfer.
			Basic discussion of transfer timeline/plan.	Good outlining of patients transition/transfer goals and timeline.
				Discusses adult vs peds cultural differences.
Comments:				
ITEM 6: ASSESSED PATIENT & PARENT UNDERSTANDING OF DISEASE AND TREATMENT				
1	2	3	4	5
Did not ask patient or parent their understanding of asthma etiology, complications, or treatment.		Asked parent or patient about their understanding of asthma and treatment.		Asked patient AND parent about their understanding of asthma and treatment.
		Added to patient's/parent's current understanding.		Effectively built-off of current understanding with meaningful education targeted to the patient and parent.
Comments:				
ITEM 7: TRANSITION SKILLS GLOBAL SCORE				
Very Poor	Inadequate	Adequate	Good	Outstanding
1 1.5	2 2.5	3 3.5	4 4.5	5
Comments:				

The faculty assessors also rated the skill of assessing the patient’s understanding of their disease; while this is important during patient encounters, it was not taught in the podcasts nor included in the OSCE station instructions and therefore served as a "control” domain. Although always good practice, this sixth skill is not specific to transition and therefore was not a focus of the podcasts or OSCE rubric. Scores that differed by more than one point during the independent scoring were reconciled by the two assessors, who were considered to have arrived at a consensus when their scores were within 0.5 points of each other. The two assessors’ mean score is reported for each resident. Pre- versus post-curriculum OSCE scores were compared using the paired, two-tailed student t-test, with p<0.05 serving as the threshold for statistical significance.

## Results

Resident participation and engagement

All six residents completed the rotation in which the curriculum was delivered, and all participated in the curriculum: five of six residents listened to all nine podcasts, while one resident instead chose to read the podcast transcripts. Unstructured feedback from the residents was collected informally to create rapid changes to the curriculum as needed. Residents reported positive feedback, stating expectations and resources were clear, the schedule was easy to follow, they “really enjoyed the curriculum,” felt that it “was not much extra work,” and described the quality of the podcasts as “excellent.” They also said that listening to the podcasts was conducive to their schedule and learning style and they would listen to longer podcasts if available. One resident stated, “The way I talk with my teenage patients in the clinic has definitely changed because of this [curriculum].”

Resident self-assessment

Across all five transition goals, residents improved their average pre-self-assessment score from 2.4 to 3.93 (p= 0.0002) after completing the curriculum. All Individual goals improved as well (Table 3).

**Table 3:** Mean Difference in Transition Goal Self-Assessment Scores Among All Participants (n=6) p-value of <0.05 was used to determine statistical significance.

Goal	Goal Topic	Pre-Curriculum Self-Assessment	Post-Curriculum Self-Assessment	P-value
1	Knowledge and skills around transition of youth with special health care needs	2.67	4.5	0.006
2	Understand and navigate the development and psychosocial aspects of transition	3	4.33	0.01
3	Understand and navigate the impact on families of insurance policies and social services as patients age into adulthood,	2	4	0.006
4	Understand and navigate educational and vocational needs of youth with special health care needs	2.33	3.83	0.001
5	Apply transition health care systems knowledge to improve transition policies	2	3	0.01

Objective structured clinical examination (OSCE)

Six residents completed the pre-OSCE and five completed the post-OSCE (Table 4). Residents improved their global score from 2.6 to 4.1 (p=0.002) and when averaging their five transition domains, increased from a mean of 2.7 to a mean of 3.4 (p=0.04). Noting that residents were less likely to ask the parent to “leave the room” (domain 3) during virtual OSCEs, we also calculated pre- and post-curriculum OSCEs using only domains 1, 2, 4, and 5, in which there was an increase from 2.8 to 3.8 (p=0.008). For individual domains, domain 1 (placing the patient in the primary role and using the parent for corroboration) achieved significance as an independent measurement, with scores improving from 3.5 to 4.6 (p=0.04). Domain 6, which served as a control, did not increase from pre-curriculum scores (1.4) to post-curriculum scores (1.7; p=0.48).

**Table 4:** OSCE Curriculum Scores; Pre (n=6)/Post (n=5) OSCE = objective structured clinical examination p-value of <0.05 was used to determine statistical significance.

Domain(s)	Domain Topic	Pre-Curriculum OSCE Score (n=6)	Post-Curriculum OSCE Score (n=5)	P-value
1	Places patient in primary role and uses parent for corroboration	3.5	4.6	0.04
2	Assesses transition skills	3.1	3.4	0.30
3	Asks parent to leave room for social history and assures patient of confidentiality	2.7	1.8	0.22
4	Assesses barriers to transition and adherence	3.2	4.8	0.15
5	Discusses transition and transfer	1.3	2.5	0.07
Average of domains 1-5	Averaging all transition skills	2.7	3.4	0.04
Average of domains 1, 2, 4, 5	Averaging all transition skills except asking the parent to “step out” given the logistic challenge of doing so in a telehealth visit	2.8	3.8	0.008
6 (control domain)	Assess patient and parent understanding of their disease and treatment	1.4	1.7	0.48
Global score	Gestalt score for overall transition performance	2.6	4.1	0.002

## Discussion

Our HCT curriculum, delivered through podcasts paired with experiential learning, improved residents’ self-assessed and observed skills for effectively addressing the HCT needs of adolescent and young adult patients. The curriculum was well received with a high completion rate. Resident's self-perceived skills improved across all goals assessed. Additionally, all five HCT skills evaluated improved, with the most significant improvement noted in residents’ ability to place the patient in the primary role, directing questions toward the young adult patient, while still involving the parent in the history as a corroborative source.

This curriculum used a flipped classroom model by combining brief didactic material with experiential learning. The podcast format of the didactics was preferred over reading or lectures, yet it is still important to provide multiple modes of teaching for various learning styles, as one participant chose to read the transcripts rather than listen to the audio. One strength of the podcast curriculum is that it can be implemented within existing clinical experiences to bolster knowledge and skills and standardize teaching across all learners.

The podcast curriculum utilized an inter-professional team to ensure the incorporation of multiple perspectives and skill sets, given that HCT's success hinges on utilizing a team-based approach. One of the strengths of the podcasts was that teaching came from general pediatricians, med-peds subspecialists, adolescent medicine specialists, mental health experts, a social worker, a parent, and other content experts with unique perspectives. Of note, while this curriculum was implemented with medical trainees, it could be easily adapted for dissemination to other health care professional training contexts, such as with advanced practice providers or social work students, as well as within a variety of medical specialties or subspecialties.

Other HCT curricula have centered on the use of a transition planning tool and case discussions, demonstrating improvement in knowledge and attitudes toward HCT [16-18]. In addition, Kaushik and colleagues used asynchronous modules and use of a portable medical summary (PMS) to improve the knowledge around HCT of pediatric residents. They demonstrated improvement in skills by increasing the number of elements in the PMS that the learner included [18], although this was an intentionally narrow focus. Our curriculum was unique in that it coupled asynchronous didactic material with activities that called for the application of the material in the clinical setting, maximizing experiential learning opportunities. Our curriculum also demonstrated improvement across a broad range of HCT skills by assessing resident performance on an HCT-specific OSCE.

Study limitations include our small sample size and assessment of residents in a single training program. In addition, self-assessments are inherently subjective, which is why we coupled self-assessments with the OSCE assessment. Given that our curriculum was implemented with PGY-3 residents, it remains unknown whether learners in earlier stages of training would have sufficient clinical knowledge to benefit to the same degree. It should also be noted that moving from an in-person (pre-curriculum) to a virtual OSCE assessment (post-curriculum) created subtle variability in our assessment method, potentially impacting trainee performance. In addition, although the pre-and post-OSCE evaluations were 12 months apart, using the same clinical scenario for both evaluations may have allowed for some improvements based in part on prior exposure to the standardized patient scenario or natural progression of skills over time. Importantly, the control skill (domain 6), did not increase significantly, which supports the idea that improvement in HCT skills is not solely, or even largely, due to repeating the OSCE or training stage-dependent improvement in clinical skills. Post-curricular OSCEs were performed between 0-5 months after the completion of the curriculum (average of 2.5 months), depending on which month the resident rotated through the curriculum. Therefore, it does not demonstrate whether skills would be retained for a year or more following the curriculum.

Finally, it is important to recognize that HCT is a complex topic with many facets, lending itself well to the combination of didactic material and experiential opportunities in which trainees can observe real-life transition challenges and practice HCT skills. It is difficult to separate the effect of experiential learning from the didactic activity as they were designed to be synergistic in accordance with Kolb’s theory of experiential learning. However, given that the self-assessments were collected after residents had completed their first exposure to the experiential activities but before exposure to the didactic curriculum, improvement in self-assessment scores points towards the importance of the didactic podcasts. In addition to providing an opportunity for formative assessment, the OSCE provided an excellent opportunity for trainees to consolidate HCT skills and receive feedback on skills mastered and skills requiring additional attention. 

Although many of the clinical experiences in our ambulatory rotation were centered on the transitioning patient in subspecialty contexts, the experiences also included primary care and other specialty clinics. All patient care areas encountering adolescents and young adults should incorporate HCT care into their practices. Our podcast series, provided for free through this publication, can be utilized by other training programs to provide structured didactic instruction on HCT, and the curriculum need not be embedded in a rotation with a specific HCT emphasis.

Future research is needed to evaluate the impact of HCT curricula on diverse trainee populations, including from other primary care specialties with larger trainee cohorts, and potentially across multiple institutions where the rotation and clinical experiences will be unique, helping to isolate the effect of the curriculum itself.

## Conclusions

In summary, we present a pilot podcast-based curriculum for teaching HCT skills as well as a novel OSCE tool for teaching and assessing HCT skills. Implementation of this curriculum by other programs does not require synchronous didactic time or onsite HCT experts, which makes this podcast series versatile and easy to employ. The podcasts and learning exercises can be incorporated into existing clinical experiences, capitalizing on the benefits of a flipped classroom strategy and enabling easy integration with existing clinical assignments. Therefore, we hope that other training programs will take advantage of the curriculum to improve trainees’ HCT skills, with the ultimate goal of improving clinical outcomes for young adult patients.

## Appendices

Appendix A: Goals & Objectives

Purpose:

To create a longitudinal curriculum that applies active and experiential learning for med-peds residents to develop the knowledge, attitudes, and skills necessary to provide transitional care support to adolescents and young adult patients and their families as they prepare to transfer from pediatric to adult care, or as they continue care post-transfer into an adult-oriented healthcare context.

Core Learning Goals and Objectives:

A.      Goal 1: Residents will be able to apply core pediatric to adult transition and transfer skills.

1.      Define transition, the key elements of transition, and describe the significance of transition for adolescents and young adults (AYA) with and without special health care needs

2.      Locate core transition resources, including a transition readiness assessment, a transition skills timeline, and a transfer plan template

3.      Administer and interpret a transition readiness assessment questionnaire to patients in their own practice

4.      Describe key cultural differences between the pediatric-centered and adult-oriented health care systems

5.      Explain transition and transfer to an AYA patient and his/her family

6.      Recognize patient-related and disease-related factors that constitute ideal timing for transfer from pediatric to adult care

7.      Identify five strategies to help a transferring AYA patient integrate into an adult clinic

8.      Demonstrate the ability to work as an effective member of an inter-professional team to support youth with health care transitions

B.      Goal 2: Residents will be able to explain how transition to adulthood is impacted by cognitive development, mental health, and psychosocial challenges for youth with special healthcare needs (YSHCN), and how chronic illness impacts educational and vocational goals and needs.

1.      Explain how adolescent and young adult development impacts self-management and self-advocacy skills

2.      Describe the impact of having a special health care need on maturation

3.      Identify the mental health needs in AYA with special health care needs

4.      Identify patients who may need assistance with guardianship or supported decision making

5.      Partner with AYA and families to develop self-care skills in a developmentally appropriate manner

6.      Discuss how young adults’ educational and vocational goals impact their health care as well as how their health impacts their educational and vocational progress; identify resources to help support patients and families reach these goals

C.      Goal 3: Residents will recognize the importance of insurance policies and social services on transitioning AYA and their families

1.      Summarize key changes to insurance status based on age during transition

2.      Explain insurance options for young adults

Supervision:

The clinical experiences for the core curriculum content will occur during the Med-Peds Ambulatory rotation, including the med-peds house officer continuity clinic and specialty clinics. The attending preceptors for those rotations will supervise the residents, and there may be opportunity to learn from other team members as well, including social workers and advanced practice providers.

The residents will receive a sample schedule at the beginning of the rotation. They can “check off” each item once completed. At the end of the rotation, they will review their readings and experiences with program leadership.
